# Special Issue “Microbial Biodegradation and Biotransformation”

**DOI:** 10.3390/microorganisms11041047

**Published:** 2023-04-17

**Authors:** Irina Ivshina, Elena Tyumina

**Affiliations:** 1 Institute of Ecology and Genetics of Microorganisms, Perm Federal Research Center, Ural Branch of the Russian Academy of Sciences, 13a Lenin Street, Perm 614990, Russia; 2 Microbiology and Immunology Department, Perm State National Research University, 15 Bukirev Street, Perm 614990, Russia

The current state of the environment is a major concern. The quality of the environment has decreased sharply due to anthropogenic pollution of natural ecosystems. Therefore, it has become increasingly urgent to find ways to prevent and neutralize eco-pollutants. Microbial biodegradation and biotransformation processes are important because they can help break down harmful chemicals into less toxic compounds, ultimately reducing the impact of xenobiotics on the environment and human health. In addition to being attractive for cleaning up environmental contamination, microbial biodegradative routes for recalcitrant compounds can also serve as sources of new catalytic reactions for application in green chemistry and white biotechnology [1,2].

In this regard, recent efforts of most researchers have mainly concentrated in the field of applied microbiology, which supports the search for rational ways of biodegradation and for effective biodegraders of new xenobiotic compounds that continuously enter the environment [3]. Their harmful effects are enhanced due to the simultaneous presence of many other active xenobiotics in the system, with varying degrees of degradability and toxicity. Understanding the mechanisms of microbial degradation and transformation of pollutants can help us develop effective strategies to remediate contaminated environments and promote sustainable development.

This Special Issue, entitled “Microbial Biodegradation and Biotransformation”, brings together a collection of articles that highlight recent advances in the field and shed light on the potential of microorganisms for bioremediation and biotransformation.

Several studies investigated the bioremediation potential of individual bacterial cultures. For instance, the studies by I. Zinicovscaia’s research group used a cyanobacterium, *Arthrospira platensis* (Spirulina), to remove rhenium and nickel from mono- and polymetallic synthetic effluents [4,5]. The authors determined the optimal growth phases of Spirulina for the most efficient recovery of metals. Moreover, the changes in the biomass of Spirulina and its biochemical composition (proteins, carbohydrates, lipids, phycobiliproteins, and contents of chlorophyll α and β-carotene) were examined.

L. Thi Mo et al. showed a high ability of *Rhodococcus erythropolis* X5 and S67 to degrade *n*-hexadecane at 10 °C (solid hydrophobic substrate) and 26 °C (liquid hydrophobic substrate) [6]. In addition, the authors showed that the presence of hydrocarbon results in the accumulation of intracellular electron-transparent inclusions (probably triacylglycerols) and changes to the hydrophobicity of the cell wall and fatty acid composition. It is interesting to note the formation of numerous vesicles and intracellular multimembrane structures, which likely play a key role in the assimilation of solid hydrophobic substrates in *Rhodococcus*.

To enhance the effectiveness of biodegradation processes, natural and artificial microbial consortia, immobilization of cells, and enzyme catalysts can be applied [7,8,9]. For instance, the study by E. Efremenko et al. aimed to develop an immobilized artificial consortium using a poly(vinyl alcohol) cyrogel as a carrier to degrade different organophosphorus pesticides (paraoxon, parathion, methyl parathion, diazinon, chlorpyrifos, malathion, dimethoate, and demeton-S-methyl) [10]. Two cultures of Gram-positive and Gram-negative bacterial cells, *Pseudomonas esterophilus* and *Rhodococcus ruber*, were selected, and their combination resulted in a 225% improvement in degradation activity. The immobilized artificial consortium demonstrated multiple uses and showed increased lactonase activity, highlighting the role of cell quorum in the synthetic biosystem’s efficiency.

X. Zheng and colleagues also studied the possibility of eliminating organophosphorus pesticides and a nerve agent using an enzyme catalyst [11]. For this purpose, a novel organophosphorus acid anhydrolase was isolated and purified from deep-sea sediment. This enzyme was highly effective against soman, dichlorvos, paraoxon, coumaphos, and chlorpyrifos, over a wide pH range and high salinity. The authors concluded that this enzyme can be used for decontamination of clothing, food, and polluted site bioremediation.

Immobilization may be an efficient method for accelerating microbial biotransformation to produce chemicals of economic value. C.J.C. Rodrigues and C.C.C.R. de Carvalho selected a marine bacterium, *Glutamicibacter arilaitensis* 232, for the conversion of benzaldehyde to benzyl alcohol, a chemical used as a precursor for producing esters in various industries [12]. Immobilization of cells in alginate improved the robustness of the biocatalyst, and a continuous flow reactor packed with immobilized cells significantly increased benzyl alcohol productivity.

A group of substances known as antiscalants are used to prevent the precipitation and production of scale-forming mineral salt crystals [13]. Seawater reverse osmosis is a commonly used desalination technique. A. Al-Ashhab and colleagues investigated if microbes found in seawater from a desalination plant could degrade popular commercially available antiscalants [14]. They conducted laboratory experiments on polyacrylate, polyphosphonate, and carboxylated dendrimers and studied how they affect microorganisms. The results suggest that antiscalants could affect bacterial diversity and community composition. The differences between the Choa I index (species richness) and the Shannon–Wiener index (abundance and evenness) could give insights into environmental perturbation and different bacterial adaptation strategies.

Azo dyes are frequently employed in the food, drug, cosmetic, textile, and leather industries. Synthetic azo dyes and their metabolites have significant health risks, including mutagenicity and carcinogenicity [15]. I.M. Kamal and coauthors selected highly efficient Direct Red 81-degrading bacterial mixtures isolated from tanning wastewater [16]. The mixed bacterial cultures showed high decolorization rates and tolerance to high dye concentrations, temperatures, pH, and salinity, with azoreductase being the main contributor to DR81 decolorization.

The remediation strategy for toxic compounds can be improved by combining biological approaches with chemical/physical treatments. A study conducted by M.M. Rossi et al. proposed a coupled adsorption and biodegradation process for trichloroethylene removal using a biofilm–biochar reactor [17]. The study found that the use of pine wood biochar effectively adsorbs trichloroethylene and supports the reductive dechlorination of *Dehalococcoides mccartyi*, indicating the feasibility of biochar for field-scale applications.

In addition to the ability to break down harmful chemicals, microorganisms can also play a role in negative biological effects, such as biodamage to buildings and other materials in various industrial and household situations. For example, M. Danilaev et al. studied the biodamage of novel polysiloxane coatings for the protection of organic glass under challenging natural conditions (tropical climate, high temperature, and high humidity) and laboratory conditions [18]. The authors found that under natural conditions, the main contribution to the biofouling of polysiloxane surfaces is from micromycetes of the genera *Aspergillus*, *Penicillium*, *Fusarium*, and *Alternaria*. Furthermore, fungi of the genera *Aspergillus* and *Penicillium* make the most significant contribution to biodestruction due to the production of organic acids. Nevertheless, the developed novel polysiloxane coating retains its optical-mechanical properties, which emphasizes the promise of its practical application.

Pharmaceuticals and personal care products are increasingly becoming a concern for the environment due to their potential adverse effects on human health and ecosystem functioning [19]. The COVID-19 pandemic has further highlighted the importance of understanding the fate and transport of contaminants, including emerging ones, in the environment [20,21,22]. Recently, Y. Yin and coauthors reviewed the fate of triclosan, an emerging contaminant commonly found in personal care products, in aquatic environments [23]. The review focused on the biodegradation of triclosan by isolated bacterial strains and microbial consortia, with a particular emphasis on specific and non-specific biodegradation enzymes involved in the process. This research can inform the development of strategies for managing the environmental impacts of emerging contaminants, including those associated with the COVID-19 pandemic.

Finally, I. Ivshina et al. investigated the biodegradation of non-steroidal anti-inflammatory drugs (NSAIDs) by actinomycetes of the genus *Rhodococcus* [24]. Their study examined the individual and combined effects of NSAIDs on cells, focusing on changes in morphometric characteristics, zeta potential, and catalase activity. This research found that the presence of NSAIDs resulted in significant changes in the morphometric and physicochemical properties of *Rhodococcus cerastii* IEGM 1278 cells, as well as a decrease in catalase activity. However, the bacteria were still able to degrade NSAIDs, suggesting that rhodococci have the potential to be used in the bioremediation of environments contaminated with NSAIDs. This research highlights the importance of understanding the effects of emerging contaminants on bacteria and the potential for bioremediation to mitigate their impacts on the environment.

Overall, the studies presented in this Special Issue, “Microbial Biodegradation and Biotransformation”, demonstrate the diversity and versatility of microorganisms in the bioremediation and biotransformation of organic and inorganic compounds and provide valuable insights into the mechanisms of microbial degradation and transformation. The findings of these studies have important implications for the development of sustainable solutions to environmental pollution and the promotion of a circular economy.
